# *Plasmodium falciparum* multidrug resistance gene-1 polymorphisms in Northern Nigeria: implications for the continued use of artemether-lumefantrine in the region

**DOI:** 10.1186/s12936-020-03506-z

**Published:** 2020-11-30

**Authors:** Auwal Adamu, Mahmoud Suleiman Jada, Hauwa Mohammed Sani Haruna, Bassa Obed Yakubu, Mohammed Auwal Ibrahim, Emmanuel Oluwadare Balogun, Takaya Sakura, Daniel Ken Inaoka, Kiyoshi Kita, Kenji Hirayama, Richard Culleton, Mohammed Nasir Shuaibu

**Affiliations:** 1 Department of Biochemistry, Ahmadu Bello University, Zaria, Nigeria; 2 Department of Biochemistry, Modibbo Adama University of Technology Yola, Yola, Nigeria; 3 School of Applied Science, Kaduna Polytechnic, Kaduna, Nigeria; 4 Institute of Tropical Medicine and Global Health, Nagasaki University, Nagasaki, Japan; 5 Institute of Tropical Medicine (NEKKEN), Nagasaki University, Nagasaki, Japan; 6 Department of Molecular Parasitology, Proteo-Science Center, Ehime University, Ehime, Japan

**Keywords:** Anti-malarial drug resistance, *P. falciparum*, Single nucleotide polymorphisms, *pfmdr1*, Haplotypes

## Abstract

**Background:**

The analysis of single nucleotide polymorphism (SNPs) in drug-resistance associated genes is a commonly used strategy for the surveillance of anti-malarial drug resistance in populations of parasites. The present study was designed and performed to provide genetic epidemiological data of the prevalence of N86Y-Y184F-D1246Y SNPs in *Plasmodium falciparum* multidrug resistance 1 (*pfmdr1*) in the malaria hotspot of Northern Nigeria.

**Methods:**

*Plasmodium falciparum*-positive blood samples on Whatman-3MM filter papers were collected from 750 symptomatic patients from four states (Kano, Kaduna, Yobe and Adamawa) in Northern Nigeria, and genotyped via BigDye (v3.1) terminator cycle sequencing for the presence of three SNPs in *pfmdr1*. SNPs in *pfmdr1* were used to construct NYD, NYY, NFY, NFD, YYY, YYD, YFD and YFY haplotypes, and all data were analysed using Pearson Chi square and Fisher’s exact (FE) tests.

**Results:**

The prevalence of the *pfmdr1* 86Y allele was highest in Kaduna (12.50%, ^2^ = 10.50, P = 0.02), whilst the 184F allele was highest in Kano (73.10%, ^2^ = 13.20, P = 0.00), and the *pfmdr1* 1246Y allele was highest in Yobe (5.26%, ^2^ = 9.20, P = 0.03). The NFD haplotype had the highest prevalence of 69.81% in Kano (^2^ = 36.10, P = 0.00), followed by NYD with a prevalence of 49.00% in Adamawa, then YFD with prevalence of 11.46% in Kaduna. The YYY haplotype was not observed in any of the studied states.

**Conclusion:**

The present study suggests that strains of *P. falciparum* with reduced sensitivity to the lumefantrine component of AL exist in Northern Nigeria and predominate in the North-West region.

## Background

Anti-malarial drug resistance is a major impediment to malaria chemotherapy in sub-Saharan Africa [1] largely because *Plasmodium falciparum* rapidly develops resistance to drugs [2]. Resistance to anti-malarial drugs occurs through drug-selection of spontaneous mutations in *P. falciparum* that confer tolerance to the drug [3]. The selection and spread of drug resistant *P. falciparum* is facilitated by the rapid genome replication rate and by a relatively high mutation rate per generation of the parasite [4, 5]. The speed of selection of mutants within parasite populations depends upon the pharmacokinetics of the drug itself and its degree of usage within a given host population [1]. For many anti-malarial drugs, molecular markers of parasite resistance are known. Surveillance of these markers in parasite populations can act as a proxy measure of the efficacy of drugs within that population, and can act as early warning signals of the emergence of resistance into new regions. Frequent and thorough molecular surveys of the prevalence of mutations associated with drug resistance can, therefore, inform regional drug policies.

Single nucleotide polymorphisms (SNPs) in the *P. falciparum* multidrug resistance gene (*pfmdr1*) have been shown to modulate the susceptibility of the parasite to the long acting partner drug in Artemisinin-based combination therapy (ACT) [6], but are not associated with resistance to artemisinin. Artemether-lumefantrine (AL) and artesunate-amodiaquine (AS-AQ) are two combinations commonly used in ACT in sub-Saharan Africa to treat uncomplicated malaria, but until now, there is no clear evidence of treatment failure from their use in the region. However, some genetic studies involving *pfmdr1* have suggested opposing selective pressures following separate use of the drugs, in which parasites harbouring N86, 184F, D1246 *pfmdr1* genotypes predominate in African countries that recommend AL as first line anti-malarial drug, whilst those carrying 86Y, Y184 and 1246Y *pfmdr1* genotypes predominate in African countries that use AS-AQ as frontline anti-malarial therapy [7].

African *P. falciparum* isolates may carry the resistant allele of *pfcrt* encoding the amino acids CVIET at codons 72–76 as well as a variety of polymorphic *pfmdr1* alleles which have originated and spread within the African continent [8–10]. The *pfmdr1* gene is a structural homologue of the mammalian multidrug resistance gene encoding a P-glycoprotein homologue-1 (Pgh1) multi-drug resistant transporter [11] and is expressed into a *Pf*MDR1 transporter located in the *P. falciparum* food vacuole.

Mutations in *pfmdr1* are associated with reduced influx of diverse anti-malarial drugs reducing their intracellular accumulation [12, 13]. Single nucleotide polymorphisms (SNPs) in *pfmdr1* are associated with resistance to aminoquinolines [14, 15]. Several codons in *pfmdr1* have been putatively linked with changes in the parasite’s susceptibility to anti-malarial drugs, but codons N86Y, Y184F and D1246Y are uniquely associated with changes in sensitivity to lumefantrine (LUM) and amodiaquine (AQ) in sub-Saharan Africa [16]. While the *pfmdr1* 86Y allele was strongly associated with chloroquine (CQ) and amodiaquine (AQ) resistance [17, 18], 1246Y alleles were shown to confer resistance to quinine (QN) and possess the capacity to *increase* the parasite susceptibility to mefloquine (MQ), halofantrine (HF) and artemisinin (ART) [19, 20]. In the study of Reed et al. [21], the sensitivity of CQS D10 parasites to CQ was not affected by transfection of the parasites with *pfmdr1* D1246Y mutation, but reduced by half, due to replacement of the mutation with a wild-type D10 *pfmdr1* sequence on a different genetic background of the parasite (CQR 7G8).

The mutant *pfmdr1* 86Y and 1246Y alleles have also been linked to reduced sensitivity to AQ, whereas the wild-type *pfmdr1* N86 and D1246 alleles are linked to reduced susceptibility against LUM [22, 23]. In Africa, the common use of AL and AS-AQ in the treatment of uncomplicated malaria has been linked with the emergence of *pfmdr1* N86Y, Y184F and D1246Y SNPs [24], and the prevalence of these mutations are frequently used for evaluating changes in sensitivity to LUM and AQ partners in artemisinin-based combinations [7]. Several studies have shown that parasites carrying a combination of *pfmdr1* N86, 184F, and D1246 (the “NFD” haplotype) display decreased susceptibility to AL and that treatment with AL can select for such a haplotype [25, 26].

Nigeria accounts for 25% of global cases of malaria and an estimated 50% of the country’s population suffer at least one episode of malaria every year, while under-five children experience an average of 2 to 4 attacks in a year [27]. *Plasmodium falciparum* is stably and perennially transmitted in all parts of the country [28], with transmission increased during the wet season compared to the dry [29, 30]. North-West and North–East Nigeria have so far been identified as hotspots of malaria in relation to the southern parts of the country due primarily to climatic and environmental conditions [31]. The North-West region of the country suffers a much higher *P. falciparum* transmission rate than the other regions including North-East Nigeria [32].

The frontline drug for malaria chemotherapy in the country was chloroquine until 2005 when it was withdrawn as a result of resistance [33]. Subsequently, the artemether-lumefantrine was recommended as the only first-line drug for the treatment of uncomplicated malaria in the regions. Unfortunately, several reports investigating molecular markers of anti-malarial resistance have suggested a massive reduction of parasite susceptibility to LUM component of AL [19, 24, 34, 35]. In Uganda, Dokomajilar et al. [34] showed a high prevalence of *pfmdr1* N86, 184F, and D1246 alleles after treatment with AL, where the pattern persists even in patients that presented with clinical failure. A few years after, Mbogo et al. [24] genotyped 982 archived samples collected during 2003–2012 for *pfmdr1* polymorphisms and reported a dramatic reduction in *pfmdr1* 86Y and 1246Y alleles over time. Similarly, the relationship between presence of mutations involving *pfmdr1* 86, 184 and 1246 codons and success of ultralow-dose mefloquine treatment was investigated in Gabon, where Mawili-Mboumba et al. [35] observed a low prevalence of *pfmdr1* N86 allele but the prevalence of 184F and D1246 alleles was above 80% each. In Tanzania, Humphreys et al. [19] observed a high prevalence of *pfmdr1* 86Y, Y184 and 1246Y in patients who failed treatment with AQ, but observed the opposite in those who failed AL treatment. The prevalence of *pfmdr1* polymorphisms in Nigeria was majorly reported from the Southern region, where a positive association between *pfmdr1* N86, F184 and D1246 alleles and clinical failure was observed [36]. In contrast, a prevalence of 62.2% and 69.0% for *pfmdr1* 86Y and F184 allele, respectively, was also reported from the region [37] which was recently followed by another survey where the *pfmdr1* 86Y and 1246Y alleles had prevalence of 24% and 18.6%, respectively [38]. Yet, there is no valid baseline data involving *pfmdr1* SNPs in both North-West and North–East Nigeria since the withdrawal of CQ and adoption of AL in Northern Nigeria. In this study, the distributions of the *pfmdr1* N86Y, Y184F and D1246Y SNPs across the North-West and-East Nigeria were investigated.

## Methods

### Description of study sites

**Fig. 1:**
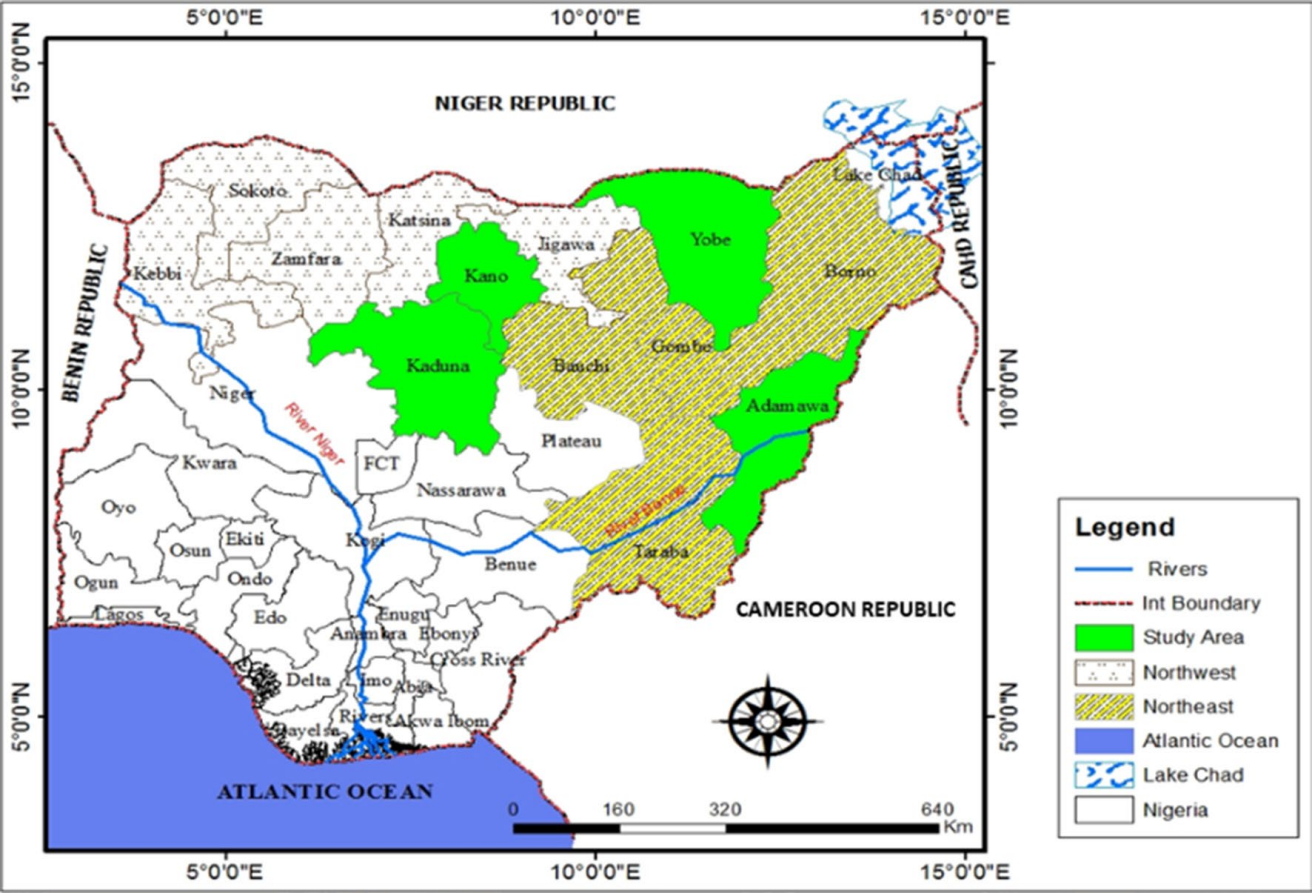
A map of Nigeria showing the study sites for the surveillance of *pfmdr1* N86Y-Y184F-D1246Y polymorphisms

Nigeria’s North-West and North–East are two out of the six geo-political zones of Nigeria. The North-West is made up of seven states and is home to a population of over 35 million people whilst the North-East comprises six states with a population of over 18 million [39]. Two states from the North-West; Kano (longitude 7° 10′ E, 10° 35′ E and latitude 10° 25′ N, 13° 53′ N) and Kaduna (longitudes 7° 23′ E and 7° 29′ E and latitudes 10° 25′ N and 10° 36′ N) with a combined population of 15,450,244 were randomly selected for inclusion in this study while Yobe (longitude 13.5° E and latitude 11° N) and Adamawa (longitude 11° and 14° E and latitude 7° and 11° N) states with a combined population of 5,489,692 were similarly selected from the North-Eastern region [39]. Other relevant details about the study sites are indicated in Fig. 1.

### Selection criteria

In this study, patients who presented with symptoms and confirmed of uncomplicated malaria across all ages, but did not take any anti-malarial drug 2 weeks previously before arrival to the facilities, were included, this is because previous treatment with any of the commonly used AL, QN and sulfadoxine-pyrimethamine (SP) anti-malarials may amplify establishment of drug resistant parasites, whilst those who presented with severe malaria were excluded.

### Sample collection

Between June and November 2017, thick and thin film microscopy was used to confirm *P. falciparum* positivity of malaria symptomatic patients that attended selected health facilities within the study sites. The total number of samples collected from Kano, Kaduna, Adamawa and Yobe were 250, 150, 150 and 200 respectively. 10 µL of microscopically confirmed *P. falciparum* parasitized blood samples were spotted on four different positions onto Whatman-3MM filter papers and allowed to dry at room temperature. Each sample was placed in sachets containing desiccant, and was preserved in a refrigerator at 4 °C.

### Genomic DNA isolation, amplification and genotyping of *pfmdr1*

Three discs (3 mm/disc) were punched from the *P. falciparum*-positive dried blood spots and the punch sterilized between each sample. The discs were used to extract genomic DNA using a QIAamp DNA Mini Kit (Qiagen Inc, Japan) according to the manufacturer’s instructions. Nested polymerase chain reaction (PCR) was carried out as described by Humphreys et al. [19] with slight modifications to the PCR cycle programs (Table 1). The nested PCR runs for the amplification of two separate *pfmdr1* segments S1 and S2, spanning codons 86-184, and -1246 respectively were performed with a 10 μL final master mixture and 200 ηM primers, and 1U ExTaq polymerase (Takara-Bio, Japan). Two µL of isolated genomic DNA was added to each of the first PCR master mixtures and run in the thermocycler. At the completion of the first PCR runs, 1 μL of each of the resulting amplified fragments were further used as templates in 10 μL of secondary PCR reactions. The PCR reactions were run along with parasite free genomic DNA (negative) and 3D7 clone *P. falciparum* genomic DNA (positive) controls. Two μL of each PCR product was evaluated by electrophoresis on 1.5% agarose gels that were stained with Midori Green Advance (Bulldog Bio Inc, USA) and visualized under ultraviolet light. The remaining 8 μL nested PCR products were stored at − 30 °C. All nested amplicons were subsequently purified using the one step ExoSAP-IT (ThermoFisher Scientific, Japan) purification kit and the resulting products subjected to BigDye Terminator (v3.1) Cycle Sequencing (ThermoFisher Scientific, Japan). Sequences were analysed using BioEdit Sequence Alignment Editor (v7.0.5.3) while *pfmdr1* SNPs were determined using MEGA5 software (Build#:5130611) in reference to the *pfmdr1* sequence of *P. falciparum* deposited at the NCBI database [Accession Number X56851]. Consequently, the samples bearing mixed genotypes were excluded in the calculations of prevalence of *pfmdr1* alleles and haplotypes. The prevalence was calculated as shown by the following formula:

$${\text{Prevalence of }}pfmdr1{\text{alleles per codon = }}\frac{\text{Number of wild or mutant alleles}}{\text{Sum of wild and mutant alleles per codon}}\ \times100$$

**Table 1:** Primers for Nested PCR of *pfmdr1* Long and Short Fragments and Cycling Programs Adapted and Modified from the Work of Humphreys et al. [19]

Primers	Amplicon sizes (bp)	PCR cycling programs
Outer forward FN1/15_- AGGTTGAAAAAGAGTTGAAC Outer reverse REV/C15_-ATGACACCACAAACATAAAT	578	34 cycles of 94 °C for 30 s; 55 °C for 30 s; and 65 °C for 1 min; then 65 °C for 5 min and 4 °C forever 34 cycles of 94 °C for 30 s; 55 °C for 1 min; and 65 °C for 1.5 min; then 65 °C for 5 min and 4 °C forever
Outer forward MDRFR2F15_-GTGTATTTGCTGTAAGAGCT Outer reverse MDRFR2R15_-GACATATTAAATAACATGGGTTC	958	
Nested forward MDR2/15_-ACAAAAAGAGTACCGCTGAAT Nested reverse NEWREV15_-AAACGCAAGTAATACATAAAGTC	534	29 cycles of 94 °C 30 s; 60 °C for 30 s; and 65 °C for 1 min; then 65 °C for 5 min and 4 °C forever 28 cycles of 94 °C for 30 s; 60 °C for 30 s; and 65 °C for 1 min; then 65 °C for 5 min and 4 °C forever
Nested forward MDRFR2F25_ CAGATGATGAAATGTTTAAAGATC Nested reverse MDRFR2R25_-TAAATAACATGGGTTCTTGACT	864	

Each PCR run was preceded by an initial denaturation at 94 °C for 3 min

### *Pfmdr1* N86Y-Y184F-D1246Y haplotypes

The *pfmdr1* haplotypes used in this study were based on eight previously reported haplotypes associated with artemether lumefantrine-tolerance; *pfmdr1* N86Y, Y184F and D1246Y single nucleotide polymorphisms in different *P. falciparum* populations from Africa [40, 41].

### Statistical analysis

All data were statistically analysed using Pearson Chi square and Fisher’s exact (FE) tests of Graph-Pad Prism (8.1.0) and P values ˂ 0.05 were considered to be statistically significant.

## Results

### Prevalence of SNPs in *pfmdr1* codons 86, 186 and 1246

**Fig. 2:**
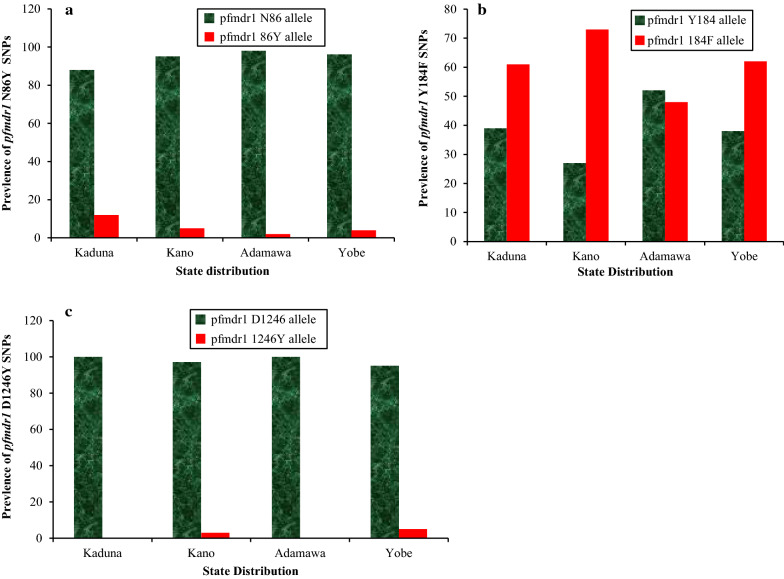
Prevalence of *pfmdr1* Single Nucleotide Polymorphisms (SNPs) across Four Stat*e*s of Northern Nigeria. The State-wise Distribution of *pfmdr1* SNPs at codons 86, 184 and 1246 are shown in A, B and C respectively. Green and red bars in A, B and C (wild and mutant alleles, respectively) are significantly different at codons 86 (^2^ = 10.50, P = 0.02), 184 (^2^ = 13.20, P = 0.00) and 1246 (^2^ = 9.20, P = 0.03) *of pfmdr1*

Of the 750 *P. falciparum* positive samples collected from the four states in Northern Nigeria, 500 were successfully genotyped for the *pfmdr1* N86Y, Y184F and D1246Y alleles, possibly due to a very low parasite density in the remaining samples. Six of the genotyped *pfmdr1* sequences were deposited in the GenBank with accession numbers MT472640, MT472641, MT472642, MT495456, MT495458, and MT495459 on the basis of presence or absence of mutations in the three codons of the gene. The prevalence of the mutant *pfmdr1* 86Y allele was observed to be significantly (P = 0.02) different across the states, with highest prevalence of 12.50% obtained in Kaduna state, followed by Kano with 4.68% and 2.00% in Adamawa state (Fig. 2a). The prevalence of the mutant *pfmdr1* 184F allele was 73.10% in Kano state, 61.46% in Kaduna state and 48.00% in Adamawa state (P = 0.00), Fig. 2b. Yobe state had the highest prevalence of the mutant *pfmdr1* 1246Y allele (5.26%) while Kaduna and Adamawa states had zero prevalence of this mutation (P = 0.03) (Fig. 2c).

**Fig. 3:**
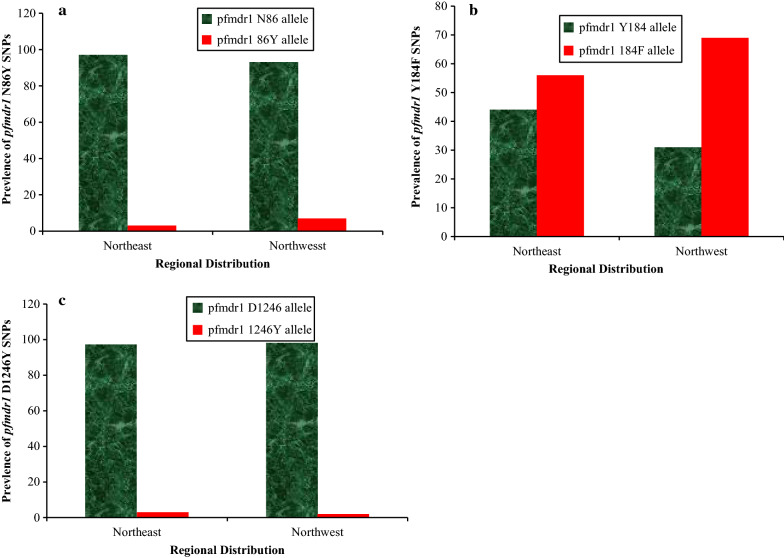
Prevalence of *pfmdr1* Single Nucleotide Polymorphisms (SNPs) across North-East and –West Nigeria. The Regional Distribution of *pfmdr1* SNPs at codons 86, 184 and 1246 are shown in A, B and C respectively. Green and red bars in A, B and C (wild and mutant alleles, respectively) are not significantly different at codons 86 (^2^ = 1.70, P = 0.19) and 1246 (^2^ = 3.60, P = 0.05) and significantly different at 184 (^2^ = 0.20, P = 0.65) *of pfmdr1*

The regional distributions of the mutant *pfmdr1* 86Y, 184F and 1246Y alleles are shown in Fig. 3. Based on the results, an overall regional prevalence of 7.49% and 3.00% for the *pfmdr1* 86Y allele was recorded in North-West and North–East Nigeria, respectively. However, the observed difference was not significant (P = 0.19), Fig. 3a. Similarly, the prevalence of the *pfmdr1*184F and 1246Y mutants were not significantly different between the North-West and North–East Nigeria, whereas the prevalence of the 184F allele differed significantly between these two regions; 68.91% and 56.22%, in the North-West and North-East, respectively (P = 0.05) (Fig. 3b). The prevalence of the *pfmdr1* 1246Y allele in the North-West and North–East Nigeria was 1.930% and 3.000% respectively (P = 0.65) (Fig. 3c).

### Analysis of *pfmdr1* haplotypes

**Fig. 4:**
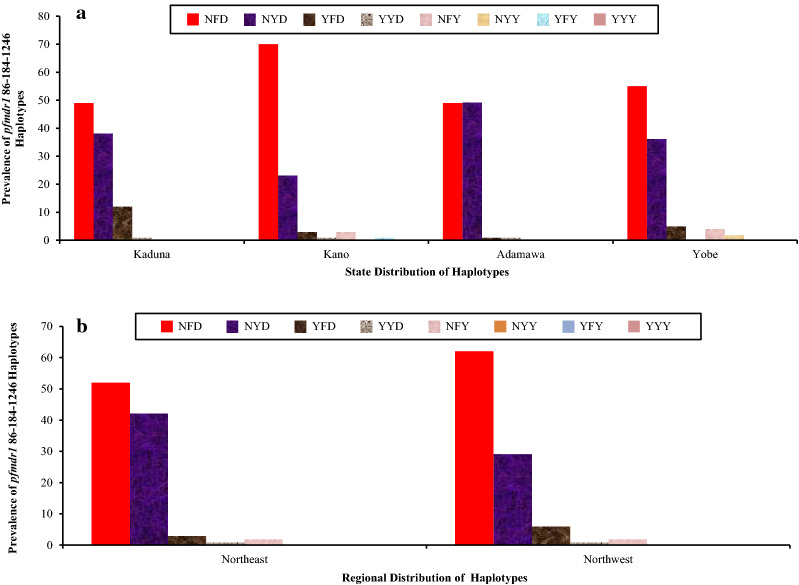
Prevalence of *pfmdr1* 86-184-1246 Haplotypes across Four States and Two Geopolitical Regions of Nigeria. The Distribution of *pfmdr1* Haplotypes across the States and Regions are shown in A and B respectively. Red, Purple, Brown, Ash, Lilac and Empty bars in A and B (*pfmdr1* NFD, NYD, YFD, YYD, NFY, NYY, YFY and YYY haplotypes, respectively) are significantly different across the states (^2^ = 36.10, P = 0.00) and not significantly different across the two regions (^2^ = 4.30, P = 0.37)

The distribution of *pfmdr1* haplotypes in the four Northern Nigerian states is shown in Fig. 4a. The *pfmdr1* N86Y, Y184F and D1246Y mutations were constructed into NYD, NYY, NFY, NFD, YYY, YYD, YFD and YFY haplotypes. Out of the 500 *pfmdr1* samples genotyped, a total of 486 haplotypes were constructed (sub-divided into seven different types). As shown in the Fig. 3, there was a significant difference in the prevalences of all *pfmdr1* haplotypes across the locations (P = 0.00); the NFD *pfmdr1* haplotype was highest in Kano state with a prevalence of 69.81%, the NYD haplotype was highest in Adamawa with a prevalence of 49.00%, whilst the *pfmdr1* YFD haplotype predominated in Kaduna with a prevalence of 11.46%. The *pfmdr1* YYY haplotype was not detected (Fig. 4a). Figure 4b showed the distribution of the *pfmdr1* haplotypes across North-West and North–East Nigeria, but in contrast to the states distribution, no significant difference was observed (P = 0.37). The *pfmdr1* NFD haplotype was highest in the North-West with a prevalence of 61.96%, the NYD haplotype was highest in the North-East with a prevalence of 41.63%, and the YFD haplotype was highest in the North-West with a prevalence of 5.88%.

## Discussion

Mutations in several genes, including *pfmdr1*, *pfcrt* and *pfk13* are associated with variation in parasite sensitivity to a range of drugs [12, 42, 43]. The *pfmdr1* mutations N86Y, Y184F and D1246Y SNPs are thought to modulate susceptibility to CQ, AL and AS-AQ [6]. It was observed that, *pfmdr1* 184F and 86Y alleles predominated in North-West Nigeria while 1246Y was higher in the North-East.

Alleles of *pfmdr1* carrying the wild type N86 residue are associated with higher IC_50_ and IC_90_ values for LMF, MFQ and DHA, while the alternative 86Y residue seems to confer increased resistance against CQ and AQ [6]. Similarly, there are varying epidemiological reports on the prevalence and consequences of *pfmdr1* N86Y polymorphisms from different parts of the world. For example, Ibraheem et al. [44] suggested that *pfmdr1* mutations are geographically confined and have inconsistent distributions from one geographic region to another.

Following adoption of ACT in many African countries, some studies from West Africa have linked the prevalence of the *pfmdr1* N86 allele to selection by AL [7]. Therefore, the high prevalence of *pfmdr1* N86 allele observed in the present study, even with the absence of clinical data of patients, might be suggestive of possible AL pressure in all the states. In addition, the finding might also suggest that the efficacy of the LMF component of ACT is susceptible to the emergence of tolerance in the local *P. falciparum* populations, as the presence of *pfmdr1* N86 is critical in the initiation of resistance to LMF in vivo and that its selection primarily follows reinfection and recrudescence events associated with the elimination stage of LMF, 4–5 days after artemether clearance [45].

Some reports have associated a rise in the prevalence of *pfmdr1* 86Y alleles with increasing CQ resistance [14, 46, 47]. The low prevalence of *pfmdr1* 86Y in Adamawa and Yobe raises the possibility that CQ may be effective against *P. falciparum* malaria in North-Eastern Nigeria once again, although this would be presumably tempered by CQ-resistance associated mutations in *pfcrt*, which was not assayed here. It is possible that the selection of *pfmdr1* 86Y allele in this region was aided by the cessation of CQ usage due to the emergence of resistance. The high prevalence of this mutation across Northern Nigeria may indicate that the efficacy of AL is at risk in this region, but raises the possibility that CQ may be effective in the chemotherapy of uncomplicated malaria here.

The *pfmdr1* Y184F polymorphisms have been reported by various in vitro studies to be weakly associated with changes in anti-malarial drug response [6, 12, 48]. The IC_50_ of some anti-malarial drugs was shown to vary on the sole acquisition of either *pfmdr1* N86 or 86Y alleles by parasite lines expressing wild type *pfmdr1*-Y184 or mutant 184F alleles [6]. Several epidemiological studies on the prevalence of *pfmdr1* Y184F polymorphisms have shown that the Y184 allele is predominantly confined to East and Central Africa while the mutant 184F allele predominates in West Africa [7, 49–51]. Okell et al. [7] observed higher occurrence of *pfmdr1* 184F in West Africa than East and Central Africa after extracting data from 397 surveys measuring the prevalence or frequency of at least one *pfmdr1* polymorphism in 30 countries. In Cameroon, the prevalence of *pfmdr1* 184F allele was shown to reduce drastically from 97.3 to 56% in 2003–2013 [50]. Achieng et al. [49] showed that between 1995 and 2003 prior to the introduction of ACT in Kenya, the prevalence of *pfmdr1* Y184 was 100%, but declined to 99.3% between 2008 and 2014 post ACT introduction. In a study conducted in Ghana by Duah et al. [52] using archived filter paper blood spots from under-five children with uncomplicated malaria in 2003–2010, the prevalence of *pfmdr1* 184F was reported to increase steadily as 26–78%, 35–82%, 48–70%, and 40–80% for 2003–2004, 2005–2006, 2007–2008, and 2010, respectively. Indeed, reports of the high occurrence of the mutant *pfmdr1* 184F in West Africa were corroborated by the present findings in which it was observed that the prevalence of *pfmdr1* 184F was high in all the states, and especially in Kano, and that its prevalence is higher in North-West compared to North-East Nigeria. This mutation has been previously linked to a reduction in susceptibility to LUM and/or ART [53]. It is perhaps unsurprising that a relatively high prevalence of this mutation was found in regions, such as Tanzania in East Africa, where AL is first-line intervention against uncomplicated malaria. Thus, the high prevalence of mutant *pfmdr1* 184F obtained in the present study may simply reflect the relatively common use of AL treatment in this region.

The *pfmdr1* D1246Y mutation affects *P. falciparum* susceptibility to various anti-malarials including QN, MFQ, (HF), CQ and ART, with the latter two drugs affected in a strain specific manner [16, 21]. The observed low prevalence of mutant *pfmdr1* 1246Y alleles compared to the wild type in this study is consistent with reports from Southern Nigeria [37] as well as other West and East African countries that adopted AL as a front-line anti-malarial therapy for uncomplicated malaria [7, 49]. Countries in Central Africa have observed an unsteady increase in the prevalence of the *pfmdr1* D1246 allele, possibly due to the selective pressure of AS-AQ [42, 54].

Several reports from Africa have suggested that linkage between *pfmdr1* N86Y/Y184F/D1246Y results in haplotypes with particular phenotypic characteristics that may be selected depending on the particular drugs that the population is exposed to [55, 56]. The occurrence of *pfmdr1* NFD and NYD haplotypes, for example, may result from AL selection while the *pfmdr1* YYY haplotype may be favoured in regions where parasites are exposed to AS-AQ, DHAP and CQ [12, 50]. The treatment of uncomplicated malaria with AL often selects *pfmdr1* haplotypes bearing the N86 allele [51, 57]. A predominance of the *pfmdr1* NFD haplotype was found in Northern Nigeria with Kano, and a complete absence of the *pfmdr1* YYY haplotype. These findings are in line with the selective effect of AL on the NFD haplotype. Hence, the abundance of *pfmdr1* NFD haplotype may suggest a loss of susceptibility to AL treatment by the parasites in the region. Resistance associated SNPs in *pfk13* should be analysed in a subsequent follow-up study, so as to confirm the influence of *pfmdr1* NFD haplotype on reduced AL sensitivity in the regions.

## Conclusions

In conclusion, Kaduna and Kano States had higher prevalences of the *pfmdr1* 86Y allele than Yobe and Adamawa indicating differential selection in North-East and North-West Nigeria, possibly due to differing population density and malaria transmission intensity in these regions. Furthermore, there was a very high prevalence of *pfmdr1* NFD and NYD haplotypes, which may suggest a reduction in susceptibility to AL in both regions. Overall, the scarcity of *pfmdr1* YYY combined with a high prevalence of NFD haplotypes suggests there is a need for continuous pharmacovigilance surrounding the use of AL in these regions.

## Data Availability

Authors assure that data will be available upon request following acceptance and publication of the article.

## Abbreviations

SNPs: Single nucleotide polymorphisms
*pfmdr1*: *P. falciparum* multidrug resistance gene-1
*pfcrt*: *P. falciparum* chloroquine resistance transporter gene
Pgh-1: P-glycoprotein homologue-1
*Pf*MDR1: *P. falciparum* multidrug resistance protein-1
DNA: Deoxyribonucleic acid
PCR: Polymerase chain reaction
CIOMS: Council for International Organizations of Medical Sciences
ACT: Artemisinin-based combination therapy
CQ: Chloroquine
AQ: Amodiaquine
QN: Quinine
HF: Halofantrine
ART: Artemisinin
MQ: Mefloquine
AL: Artemether-lumefantrine
AS-AQ: Artesunate-amodiaquine
DHAP: Dihydroartemisinin-piperaquine
